# RECEIPT OF GENDER-AFFIRMING SURGERY AMONG MEDICARE BENEFICIARIES

**DOI:** 10.1093/geroni/igad104.1361

**Published:** 2023-12-21

**Authors:** Em Balkan, Gray Babbs, Theresa Shireman, Jaclyn Hughto, David Meyers

**Affiliations:** Brown University School of Public Health, Providence, Rhode Island, United States; Brown University, Providence, Rhode Island, United States; Brown University, Providence, Rhode Island, United States; Brown University, Providence, Rhode Island, United States; Brown University, Providence, Rhode Island, United States

## Abstract

While transgender and gender diverse (TGD) people may never seek treatment related to their gender identity, many require access to gender-affirming treatments, including surgery. Starting in 2014, Medicare began covering gender-affirming surgery (GAS) on a case-by-case basis. In this study, we use national Medicare data to compare receipt of GAS by beneficiary characteristics. We classified TGD beneficiaries using an algorithm that combines diagnosis and procedure codes. Our study compared characteristics for all TGD identified beneficiaries and those who received GAS using a population-averaged logistic model using a generalized estimating equation (GEE) adjusting for dual status, age group, race/ethnicity, original reason for entitlement, and CMS region. Our sample accounted for 49,945 observations for 11,162 individuals, with a total of 290 identified records of GAS. In our adjusted analysis, we found that TGD beneficiaries in certain parts of the country had lower odds of GAS compared to their counterparts. Compared to CMS Region 1 (which includes Connecticut, Maine, Massachusetts, New Hampshire, Rhode Island, and Vermont), those living in Region 3 (Delaware, District of Columbia, Maryland, Pennsylvania, Virginia, and West Virginia), Region 4 (Alabama, Florida, Georgia, Kentucky, Mississippi, North Carolina, South Carolina, and Tennessee), and Region 6 (Arkansas, Louisiana, New Mexico, Oklahoma, and Texas) had a significantly lower odds of receiving GAS. Access to gender-affirming care may be influenced by CMS regional contractors, as well as state-by-state variation in Medicaid coverage for gender-affirming care. Further research is needed to explain why beneficiaries may have lower access to GAS to inform policy recommendations.

